# Mode Visualization and Control of Complex Lasers Using Neural Networks

**DOI:** 10.1021/acsphotonics.5c01710

**Published:** 2025-09-09

**Authors:** Wai Kit Ng, T. V. Raziman, Dhruv Saxena, Korneel Molkens, Ivo Tanghe, Zhenghe Xuan, Pieter Geiregat, Dries Van Thourhout, Mauricio Barahona, Riccardo Sapienza

**Affiliations:** † Blackett Laboratory, Department of Physics, 98992 Imperial College London, London SW7 2BW, U.K.; ‡ Department of Mathematics, 4615 Imperial College London, London SW7 2AZ, U.K.; § Physics and Chemistry of Nanostructures (PCN), 26656 Ghent University, Krijgslaan 281-S3, Gent B9000, Belgium; ∥ Center for Nano- and Biophotonics, Ghent University, Ghent 9052, Belgium; ⊥ Photonics Research Group, Ghent University - imec, Technologiepark-Zwijnaarde 126, Ghent 9052, Belgium

**Keywords:** complex laser systems, mode visualization, quantum dots, lasing control, multilayer perceptron

## Abstract

Visualizing the behavior of complex laser systems is an outstanding challenge, especially in the presence of nonlinear mode interactions. Hidden features, such as the gain distributions and spatial localization of lasing modes, often cannot be revealed experimentally, yet they are crucial to determining the laser action. Here, we introduce an experimental lasing spectroscopy method that visualizes the gain profiles of the modes in a complex, disorderly coupled microring array laser using an artificial neural network. The spatial gain distributions of the lasing modes are reconstructed without prior knowledge of the laser device. We further extend the neural network to a tandem neural network that can control the laser emission by matching the modal gain/loss profile to selectively enhance the targeted modes. This mode visualization method offers a new approach to extracting hidden spatial mode features from photonic structures, which could improve our understanding and control of complex photonic systems.

## Graphical abstract

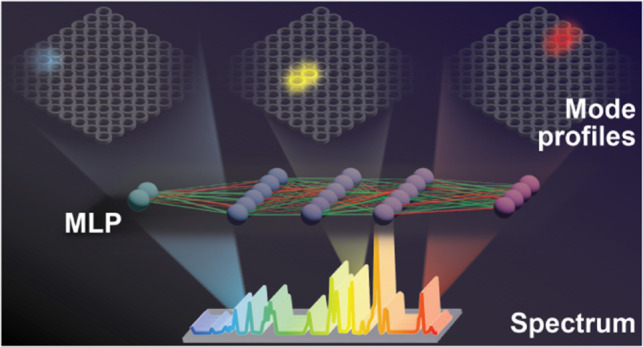

## Introduction

Complex photonic structures are feature-rich in both their material architecture and their optical response. Complex laser systems, such as random lasers,^1–5^ wave-chaotic cavities,^6,7^ and coupled lasers^8–10^ exhibit properties including a broad spectrum and low coherence. These properties cannot be easily replaced by conventional lasers^11^ and are favored in applications such as sensing^12,13^ and speckle-free imaging.^14,15^ However, the complexity and nonlinearity of these structures make their emission spectra and profiles challenging to predict and control.

Coupled nano- and microlaser systems bring together simple resonator units to form rich modes with nontrivial laser dynamics.^9,10,16–21^ Yet, these laser systems are challenging to understand and control using existing methods. Simulating the optical modes of coupled lasers would be computationally infeasible except for designs with strong symmetries,^8,22^ but adhering to these designs would inevitably reduce the functionalities of the lasers. The common method to estimate mode profiles experimentally through hyperspectral emission images would only capture far-field mode patterns and be accurate for simple lasing structures.^23^

It is possible to control lasers to output a single-mode emission with a limited tunable range by adding additional cavities to a coupled laser^9^ or by unbalancing system excitations.^8,24,25^ However, it is still challenging to systematically perform lasing control over a wide frequency range because of the lack of a simple analytical solution for these disordered and symmetry-broken structures. All of these limitations call for new methods to study and control complex laser systems.

Recently, machine learning (ML)^26^ has demonstrated the potential to provide new insights into complex systems and inverse design capabilities without requiring a complete physical model. In photonics, ML has been extensively used in the optimization of complex systems, especially for device design.^27–30^ Going beyond “black box” operation, there is growing interest in understanding how machine learning can teach us new physics in different fields, as demonstrated by studies of explainable artificial intelligence (XAI) and feature visualization in computer science, machine vision, and spectroscopy.^31–33^ Yet, the use of machine learning to understand complex lasers is still largely unexplored. In the study of complex lasers, the sophisticated processes and features that are currently experimentally inaccessible can be revealed by unfolding an ML model.

Here, we use ML to help unveil the spatial and spectral features of a complex laser system. In particular, we train and unfold an ML network model to reveal the spatial profile of the lasing modes in a disorderly coupled microring array and control its lasing spectra. The spatial gain profiles of the lasing modes are identified by reverse engineering the connection weights in a multilayer perceptron (MLP) model. Having produced a picture of the individual gain distributions of the modes, we then extend the MLP into a tandem neural network (TNN) that enables spectral control in single- and dual-mode emissions by effectively suppressing unwanted modes. Our results in mode profile visualization and selection extend the use of ML in photonic systems beyond design optimization, gaining a better understanding and control of photonic devices.

## Results and Discussion

Silicon nitride (SiN) microdisks with quantum dots (QDs) have previously been shown to be multimode resonators with a high Q factor and immense design flexibility.^34^ On the same hybrid QD-SiN platform, we fabricated a microring array with 10 × 10 microrings as a disorderly coupled laser system^34,35^ (Figure 1a, see Methods). To realize a weakly coupled complex laser system with unpredictable emission, the microrings were designed to have randomly different diameters and ring-to-ring gaps to achieve nonuniform resonance frequencies and coupling strengths (see Note S1) across the structure. In cases where the adjacent microrings have similar resonance frequencies (diameters) in proximity, the microrings can couple evanescently, resulting in collective modes that compete for gain with each other. Compared to a single microring under uniform illumination (Figure S1b), the randomness and the gain competition in the microring array result in a complicated and different lasing spectrum with more than 25 distinct lasing peaks within a 10 nm spectral range (Figures 1b and S2).

**Figure 1:**
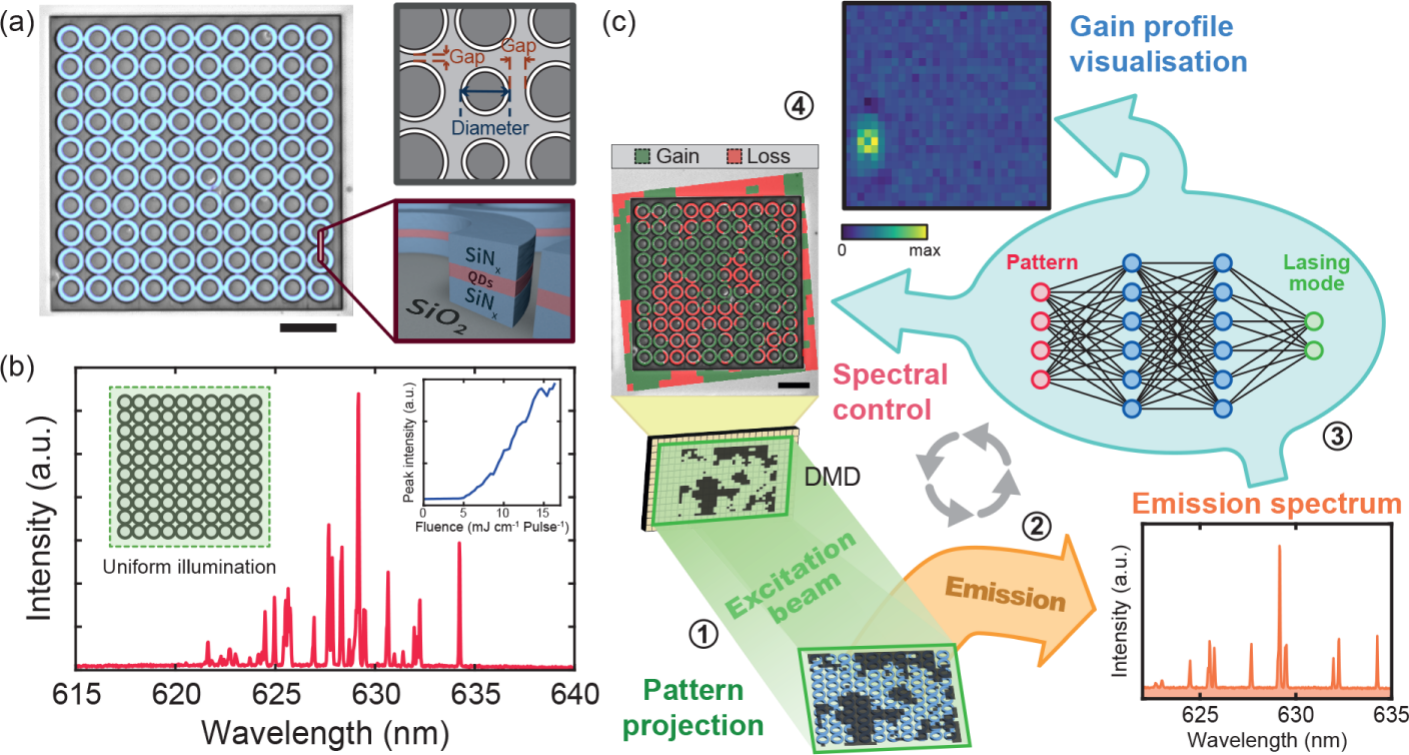
Uncovering complex lasing modes with neural networks. (a) The pseudocolor bright-field image (left) and schematic sketch (top right) of a disorderly coupled 10 × 10 QD-SiN microring array with random gaps and diameters. For detailed characteristics of the structure and material, see Figure S1. The cross-section of the microring structure (SiN/QD/SiN stack on SiO_2_ substrate) is illustrated in the bottom right. (b) The lasing spectrum of the disorderly coupled laser array under uniform excitation at a pumping fluence of 10.24 mJ cm^–2^ pulse^–1^, which shows a complex and multimode lasing behavior. The inset shows the plot of peak emission intensity under different excitation fluences with a clear lasing onset. (c) The ideas of gain profile visualization and spectral control through artificial neural networks. (1) The excitation beam, patterned via a digital micromirror device (DMD), first illuminates the microring array. (2) The selectively excited array exhibits a different gain/loss distribution for each excitation pattern, resulting in distinct emission spectra. (3) The emission spectra are then used to train the neural network model. (4) The modal spatial gain profile can be visualized by unfolding the neural network model. The color represents the intensity of the modal gain profile. Meanwhile, the control of laser emission can be achieved by solving the inverse problem through a tandem neural network. The excitation pattern, with the pumped regions in green providing gain and the unpumped regions in red causing loss, can be predicted and applied to the device to generate a particular emission spectrum. Scale bars are 20 μm.

In complex laser systems, modes exhibit distinct spatial distributions due to modal gain competition. The lasing emission can therefore be manipulated by tuning the gain profile of the system through spatially patterned excitations.^5,25,36–38^ By selectively illuminating the coupled microring array with different excitation patterns via a programmable digital micromirror device (DMD), the excited QDs embedded in the selected regions provide gain, while the remaining regions are lossy due to QD absorption (see Methods), effectively mapping the excitation pattern to the system’s gain distribution. This patterned input allows for the postfabrication modulation of the system, i.e., to selectively enhance and suppress coupled modes in a system with a fixed topology. A model capable of learning via a neural network would be able to predict the gain distributions of each lasing mode in disordered systems.

To visualize and control the lasing modes, neural network models were built to connect the gain distribution (spatial) to the lasing emission spectra (spectral) (Figure 1c). The neural networks, trained with both excitation patterns and emission spectra, can be unfolded to extract the modal gain profiles. With the gain profiles of each mode (i.e., each peak in the spectrum), the illumination pattern required to generate a particular spectrum (with a combination of modes) can be inferred by solving this inverse problem with a two-step tandem neural network. This can be achieved without extra effort to acquire additional experimental training data.

The central idea behind visualizing the gain profile of the modes is to reveal the modes’ spatial information as encoded in a trained nonlinear MLP model. The contributions of spatial pixels to a mode can then be traced back via the inner connections between layers. For simplicity, consider first a single-layer neural network model, which can be written in matrix form as

1 M=σ(W·P+B)

where **M** = [*m*_1_, ..., *m*_*M*_] is the vector of one-hot encodings for *M* modes (*m*_*i*_ ∈ {0,1}), **P** = [*p*_1_, ..., *p*_*I*_] is the vector containing the intensities of the *I* spatial pixels $\left(p_{i}\in R\right)$ , **B** is a bias vector, and σ(·) is a nonlinear activation function (e.g., sigmoid or ReLU). The nonlinearity of the activation function is the key to building a powerful and accurate model that maps the input (pixel intensities) to the output (the lasing state of a mode). Yet, for the same reason, the activation function also introduces noninvertibility, so that the spatial gain profile cannot be directly obtained from the spectrum.

In our system, however, the gain profile for each lasing mode can be estimated by considering how influential each spatial pixel is just below the lasing threshold of the mode. For a pixel that overlaps well with the mode, a small change in pixel intensity could significantly alter the state of the mode, taking it above the lasing threshold. In contrast, if the pixel does not overlap with the mode (i.e., it does not contribute to the gain), whether the mode lases or not would be irrelevant to that pixel’s intensity.

To see how a selected mode *m*_*i*_ changes with respect to changes in the intensity of pixel *p*_*j*_, it follows from eq 1 that

2 $$\frac{\partial m_{i}}{\partial p_{j}}=\frac{\partial \sigma \left({\mathrm{m̃}}_{i}\right)}{\partial {\mathrm{m̃}}_{i}}W_{\mathrm{ij}}$$

where m̃_i_ = ∑_*j*_ **W**_**ij**_ *p*_*j*_ + *b*_*i*_ and **W**_**ij**_ is the weight matrix element. For a multilabel, single-class classification neural network model, which is the type of model used in this work, the partial derivative $\frac{\partial \sigma \left({\mathrm{m̃}}_{i}\right)}{\partial {\mathrm{m̃}}_{i}}$ would then be independent of *p*_*j*_. Instead, it would only be determined by how close the system is to the threshold of mode *m*_*i*_. As the system populates closer to the threshold, the contribution of pixel *p*_*j*_ becomes more significant due to the increase in $\frac{\partial \sigma \left({\mathrm{m̃}}_{i}\right)}{\partial {\mathrm{m̃}}_{i}}$ , i.e., the system becomes more sensitive. Therefore, for the target mode *m*_*i*_, the partial derivative term is constant across each pixel *p*_*j*_, but the weight matrix element **W**_**ij**_ differs between pixels. As a result, the trainable weight matrix element **W**_**ij**_ becomes the only important factor left to distinguish how influential each spatial pixel is compared to the others, leading to visualizing the gain profile of mode *m*_*i*_.

This relationship extends to neural networks with more than one layer (Note S2) provided that the neural network has local activation functions (e.g., sigmoid or ReLU). However, for deeper networks with a large number of layers, the assumption that all relevant intermediate activation functions in the hidden layers activate simultaneously might not be satisfied, thereby increasing inaccuracies in determining the gain profile. Based on experience, our approach works best for relatively shallow MLPs with 3 or fewer layers.

To visualize the spatial gain profile of the disorderly coupled array, we trained a spectral prediction network (SN), an MLP with 2 hidden layers (1024 and 64 nodes, respectively), as shown in Figure 2a (see Note S3). To train the network, we collected the lasing responses from the array using a set of 7000 Perlin noise illuminations.^39^ The Perlin noise patterns, with some degree of clustering and sizes similar to or larger than the size of a ring, are projected through a DMD and cover the entire area of the array to sample across different gain-loss distributions (see Note S4). The experimentally collected excitation patterns and lasing spectra are then used as matched pairs to train the SN offline (Note S5). During training, the collected patterns and spectra are divided into batches to train the weight matrices **W**_**HO**_, **W**_**HH**_, and **W**_**IH**_ with the stochastic gradient-based optimization method (Adam). The values of the weight matrices are updated through backpropagation by calculating the gradient of the binary cross-entropy loss function. The model is trained when the weight matrices are optimized to correlate the input profiles with the output spectra (see Note S6).

**Figure 2:**
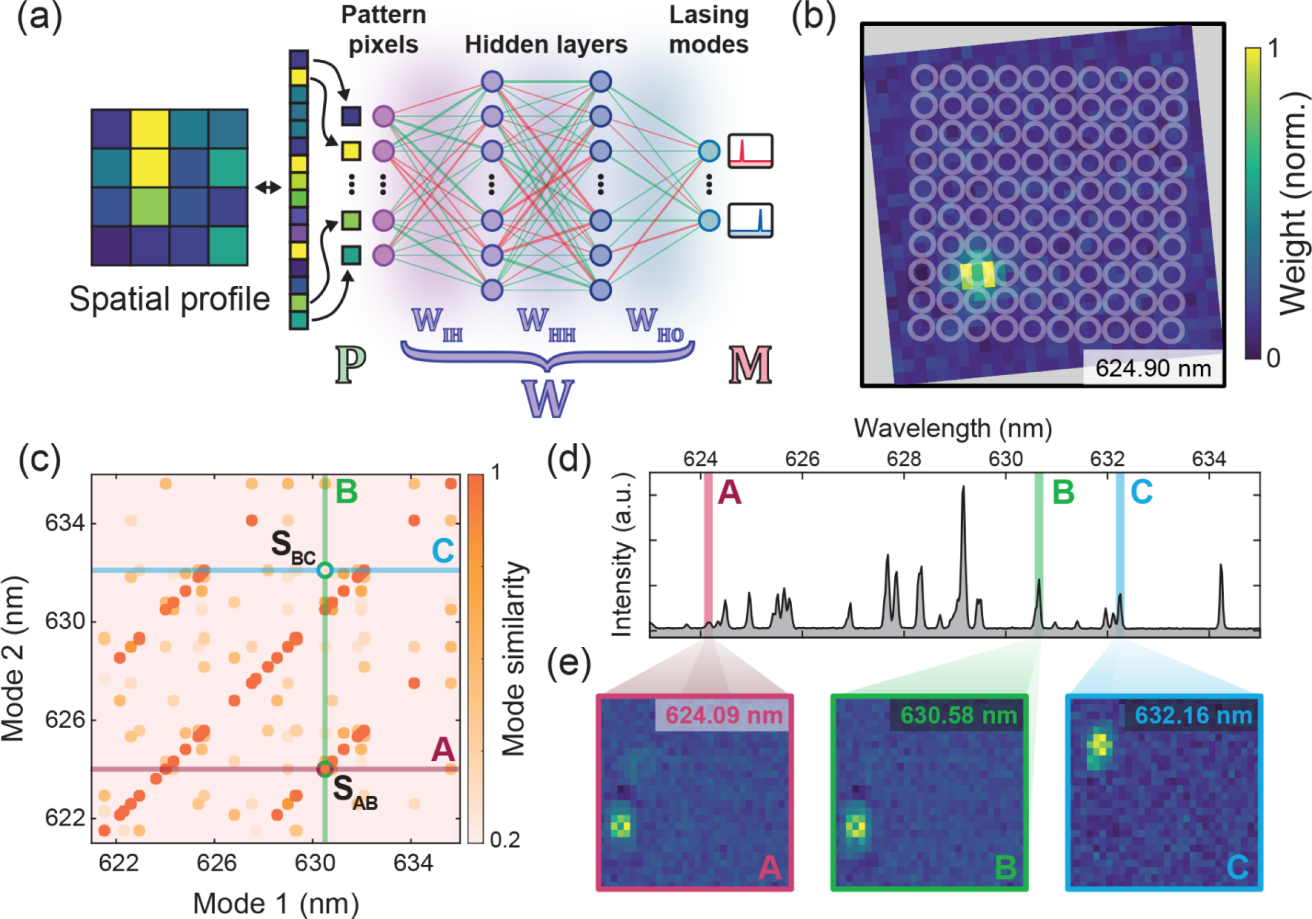
Mode visualization using a multilayer perceptron. (a) The mode visualization scheme via multilayer perceptron (MLP) neural network. The network connects the excitation patterns to emission modes. The spatial gain profile **P** of each mode can be estimated from the network weight matrices (**W** = **W**_**HO**_ · **W**_**HH**_ · **W**_**IH**_). (b) A visualized gain profile for a lasing mode at 624.90 nm with the microring sample structures superimposed. (c) The spatial similarity map between all the gain profiles of the lasing modes. The highly similar mode pairs form super- and subdiagonal lines representing a constant spectral distance between the modes. Two pairs of modes (AB and BC) with high and low similarities are also highlighted. (d) Three spectrally uncorrelated modes (A, B, and C) are highlighted in the lasing spectrum of the disorderly coupled microring array. Their spatial mode profiles are displayed in (e). The similarity between the modes can be found by crossing the two corresponding lines in (c). This shows a high similarity between modes A and B (S_AB_ = 0.9577), but not between modes B and C (S_BC_ = −0.0473).

From the trained network model, we obtain the overall weight matrix (**W** = **W**_**HO**_ · **W**_**HH**_ · **W**_**IH**_) that allows us to visualize the spatial gain profiles of different lasing modes, as illustrated in an example of the reconstructed mode at 624.90 nm (Figure 2b). Note how the recovered gain profile shows a localized circular structure that matches a microring resonator. This also aligns with our expectations of the mode distributions in the array (see also Figure S8 for the comparison with the far-field hyperspectral mode images). Due to the weak coupling in the highly random and disordered microring array, the lasing modes with the lowest threshold are likely to be those that are similar to the whispering gallery mode and localized in a single ring. This example demonstrates how using a simple neural network model enables us to visualize the underlying spatial profile of lasing modes without directly collecting spatial images of the emission.

Using this ML method provides us with a new approach to visualize the spatial modal gain profiles of laser systems. Figure 2c shows the spatial similarities of all the lasing modes in a microring array, as visualized from the MLP model. Many pairs of modes in the array exhibit a high degree of spatial overlap with a constant spectral distance between them (i.e., the super- and subdiagonal bright orange points in Figure 2c). To illustrate the relationship between mode pairs, two pairs of modes (AB and BC) are highlighted with their recovered spatial profiles (modes A, B, and C) in Figure 2e. From the multimode emission spectrum of the array (Figure 2d), it is difficult to determine whether these modes are related without knowledge of the array structure. However, in their spatial maps, mode A (624.09 nm, magenta) is nearly identical to, and colocalized with, mode B (630.58 nm, green) with a cosine similarity of 0.9577, but not with mode C (632.16 nm, blue). This indicates that modes A and B share the gain in the same microring and are therefore likely to be spatially colocalized. Indeed, the spectral separation between the modes matches the free-spectral range of a whispering gallery mode (WGM) for a 10 μm microring resonator (Δλ = 6.49 nm) (Figure 2d). Mode A is therefore very likely the higher-order mode of mode B, which is primarily located in the same resonator. This result also implies that despite being coupled, the WGMs in our microring array are only weakly perturbed due to the large detuning of resonance frequencies and weak local coupling strengths caused by structural disorder.

Beyond the visualization of modes as described above, the spatial information captured by the neural network can be used to control the laser emitting at certain modes. However, illuminating the system with a visualized spatial profile allows the excitation of modes by providing them with maximum gain; it does not guarantee the suppression of other modes—undesired modes that have a high degree of spatial overlap with the target mode might still emerge. Furthermore, the overall mode profiles for multimode lasing could differ from the sum of individual single-mode profiles—a nonlinear effect due to complex gain competition in the coupled system. Therefore, to enable the control of the laser system, it is preferable to develop a model that can predict the excitation profile directly from the lasing spectra with consideration of gain competition.

Finding an illumination profile that gives rise to a desired lasing spectrum is an inverse problem that we solve here by extending the SN into a tandem neural network (TNN). The TNN is built on the original SN with another MLP, the control network (CN), joined at the front (Figure 3a). The CN consists of three hidden layers (64, 1024, and 1024 nodes, respectively), where spectral modes are the inputs and illumination patterns are the outputs. Hence, the CN acts as a predictor for the excitation profile from a desired mode (or a combination of modes) based on the knowledge established in the SN. The SN and CN are then trained with the same set of experimental data in sequence (see Note S7). Note that both the training and test data sets are well-separated before the SN and CN are trained to avoid data leakage.^40^ After training, the CN becomes a standalone model capable of controlling the lasing mode by generating the required excitation patterns. In Figure 3b,c, we demonstrate the control of various single-mode emissions from the microring array by exciting the array with the generated patterns. Several single-mode emissions are achieved, with one exhibiting the highest side-mode suppression ratio (SMSR) of 14.41 dB at 628.20 nm. Note that, compared to the mode visualization scheme with the SN (Figure 2e), the CN does not provide high-resolution spatial profiles that recover the fine structure of the modes. Instead, the CN focuses on enhancing the target mode while suppressing the side modes, thus resulting in slightly different, more extended excitation patterns.

**Figure 3:**
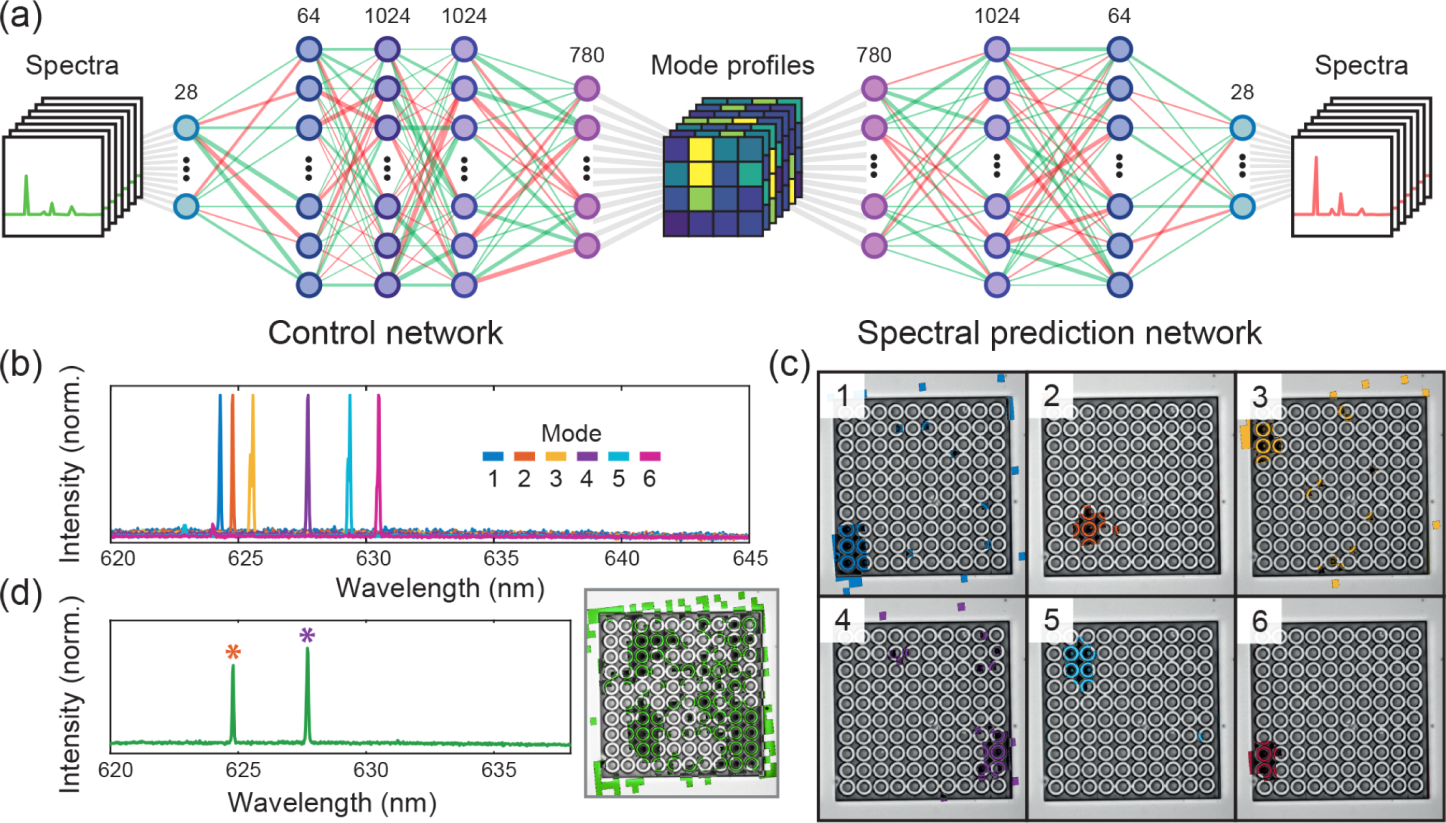
Lasing control using a tandem neural network (TNN). (a) Architecture of the TNN, which combines two artificial neural networks—the control network and the spectral prediction network—for lasing control. The number of nodes for each layer is shown at the top of the layer. In the lasing control task, the control network stands alone as a model to predict the excitation pattern required for the targeted modes after training. (b) Controlled single-mode emissions on the disorderly coupled microring array, with the highest side-mode suppression ratio (SMSR) reaching 14.41 dB. (c) The predicted excitation profiles for different single-mode emissions in (b). The target lasing modes of the spectra are (1) 624.41 nm, (2) 624.90 nm, (3) 625.67 nm, (4) 627.78 nm, (5) 629.44 nm, and (6) 630.58 nm. (d) Dual-mode (624.90 and 627.78 nm) lasing control performed on the same system using the same model. The asterisks indicate the spectral positions of the target modes. The right panel shows the corresponding predicted illumination pattern, which is complex and not equivalent to the sum of two corresponding single-mode profiles (modes 2 and 4 in (c)).

Apart from single-mode emissions, multimode emission can also be controlled using the same inverse neural network model. We demonstrated the realization of dual-wavelength lasing at 624.9 nm and 627.7 nm from the same array using the same predictor with no extra training (see Figure 3d). As a result of the gain competition among the modes, the predicted excitation profile differs from the linear sum of the two individual mode profiles (mode profiles 2 and 4 in Figure 3c).

As an attempt to enable lasing control, an iterative method was proposed in earlier work.^41^ This method iteratively adjusts the pump profile to adapt it in order to approach the target spectrum. When considering single-target optimization (i.e., only optimizing to a particular mode), our ML method requires a few thousand samples to train the entire neural network, whereas the iterative method using a genetic algorithm requires only a few hundred samples. However, for applications that frequently need to optimize across a wide variety of targets, the universality of our neural network method offers the advantage of effortlessly inferring results without requiring additional training. The iterative method, on the other hand, would necessitate individual iterative processes for different spectral targets, which would grow quickly in both the time and the number of exposures needed to optimize for multiple spectra.

Apart from universality, our lasing prediction method based on the mode visualization MLP model also enables lasing control in multiple frequencies and multimode operations. This would be a desirable feature for applications that require the simultaneous manipulation of lasing peaks, such as remote sensing via differential absorption LIDAR.^42^ Since the training and inference of the neural network can be done fully offline after training data acquisition, controlling complex lasers with this approach could enable fast switching between different sets of modes down to a few picoseconds (limited only by the carrier relaxation time) by modulating the same laser system with predefined excitation patterns. For sensing applications, a quick and more accessible selection of mode combinations could potentially improve detection accuracy by efficiently searching and for optimizing the best multimodal sensing spectrum.

Meanwhile, for visualizing complex lasing structures, previous studies have used other spectroscopy methods such as far-field hyperspectral microscopy,^43^ which estimates the mode spatial profiles by carefully imaging part of their emission and physically scanning through the whole structure. In contrast to the hyperspectral approach, our mode visualization method does not collect spatial information from the emission and only retains spectral information. Our method is not heavily affected by the emission directionality or the light scattering angles as long as the emission can be captured. This allows our method to be applied to any laser or ASE structure, regardless of the outcoupling of emission.

It is also worth mentioning that the visualization and lasing control scheme is not limited to optically pumped systems; the scheme could be transferred to electrically pumped systems as well. The modulation of gain in the system can be achieved either optically (using a patterned optical pump beam, as described in this paper) or electrically. With a mesh of local electrical contacts, the device can be selectively excited by local carrier injection, just as with a patterned optical pump. Although conceptually feasible, there are still practical barriers to demonstrating the scheme on an electrically pumped device. The difficulty of fabricating multiple small electrical contacts and the high complexity of the array design for the contacts remain major challenges waiting to be addressed.

Despite having demonstrated the visualization of coupled modes in the disordered microring array with a complex and asymmetric structure, it is still challenging to recover the mode profiles of strongly coupled structures, such as network random lasers,^25^ whose lasing modes widely overlap with each other. The efficiency of the lasing control is expected to drop as the complexity and number of modes increase. This is due to highly nonlinear behavior from strong mode competition, for which a shallow MLP model (with three hidden layers or fewer) would not be complex enough to correlate the spatial excitation profiles to their lasing spectra. Distinguishing between highly similar mode profiles also requires higher-resolution excitation patterns, which, together with a deeper network, demand significantly more lasing emission training data to uncover mode profiles. Additionally, the ML approach relies on consistent and reproducible experimental data sets. Therefore, lasing materials that change over time, such as laser dyes that gradually degrade under illumination, would limit the prediction accuracy. Stable gain media, such as semiconductors and embedded colloidal QDs, would thus be more suitable for mode visualization and lasing control with the proposed ML methods. Apart from the lasing device, the stability of the experimental setup also plays an important role in obtaining a consistent data set for both training and inference of the model. The power fluctuation of the pump (±3%) and the drift of the sample (∼0.4 μm/h) are the two major sources of setup instability that limit the size of the data set. To further improve this scheme, compensation for the sample drift and power with an active feedback mechanism could allow for more stable, large data set training. Additionally, training the neural networks with simulation data, if a good physical model is available,^25^ could be an alternative strategy that would reduce device degradation and speed up the training process.

## Conclusion

In conclusion, we have shown how artificial neural networks can visualize the modal spatial gain profiles and be used to control the lasing modes from a disorderly coupled microring array. By training the neural network with random excitation patterns, we can map the spatial gain profiles encoded in the network by unfolding the connections between neural network layers. We also demonstrated lasing control using tandem neural networks, achieving a wide range of single-mode and dual-mode emissions from the same device without extra data collection. Our work provides insight into the possibility of developing spectroscopic tools to understand the hidden features in complex laser systems, with potential applications in optical information processing, smart illumination, and optical computing.

## Methods

### Microring Array Fabrication

The disorderly coupled microring array was fabricated as reported in ref. 34. A bottom layer of 130 nm thick SiN was first deposited on a SiO_2_ wafer through low radiofrequency plasma-enhanced chemical vapor deposition (PECVD). Then, a layer of 55 nm thick CdSe/CdS quantum dots (QDs) was spin-coated. The QDs were purified and dispersed in toluene, with the concentration adjusted to achieve the desired QD layer thickness. After that, a 105 nm thick SiN top layer was deposited on the QDs as a crack-free encapsulation layer via a mixed radiofrequency PECVD process. Subsequently, photolithography was employed to define the microring structures by patterning the resist on SiN as a mask. Finally, a specifically optimized reactive ion etching (RIE) process based on a CF_4_/H_2_ gas mixture was used to etch the SiN/QD/SiN layers to attain microring arrays with smooth and steep sidewalls.

### Selection of Lasing Structure and Pixel Resolution

To demonstrate the mode visualization and lasing control with ML models, the microring array structure with the most distinct lasing modes was selected by systematically screening with selective excitations among the arrays with random sizes and gaps between the rings. This was done by switching a random pixel with different pixel sizes in an excitation pattern and observing the greatest change in the lasing responses from the microring arrays with different parameters. Due to the constraint of photobleaching occurring in the QDs under long photoexcitation, the pixel resolution was chosen as about 9 pixels per ring to balance the resolution of the visualized gain profile and the required complexity of the ML models (i.e., the number of exposures needed for the training), while maximizing the spectral changes caused by a single pixel. It is worth noting that the DMD pattern we projected was not aligned with the microring array (with an unintentional ∼6° tilt), so different rings had a slightly different overlapping shape in the pump pattern. This increased the difficulty for the ML model to infer the shapes and locations of the modes without learning.

### Optical Experiment

The microring arrays were gain-modulated and characterized in an optical microscopy setup via selective excitation (see Figure S9). The excitation source, a Nd:YAG pulsed laser (TEEM Power-Chip, λ = 532 nm, pulse width 400 ps, energy per pulse 20 μJ), was patterned with a programmable digital micromirror device (DMD, Ajile AJD-4500) and projected onto the sample through a Nikon Ti microscope mounted with a 20× objective lens (Nikon CFI Super Fluor 20×, 0.75 N.A., 1.0 mm WD). The laser beam was initially expanded by a 3× telescope to overfill the DMD to achieve a uniform excitation fluence for the pixels. For any partial illumination patterns, the overall excitation fluence delivered on the sample was held constant (10.24 mJ cm^–2^ pulse^–1^), and the DMD micromirrors were grouped into superpixels with an area of (4 × 4.7) *μ*m^2^ each. The total energy delivered to the sample was proportional to the total area of the pattern. The lasing emission from the microring array was then collected through the same objective lens, filtered, and further focused by a cylindrical lens into a vertical stripe on the spectrometer entrance slit to maximize the signal-to-noise ratio. The lasing spectrum was spectrally analyzed with a grating spectrometer (Princeton Instruments Isoplane-320) equipped with 1800 grooves/mm holographic grating (0.05 nm resolution) and a charge-coupled device camera (CCD, Princeton Instruments Pixis 400).

## Supplementary Material



## Notes

SiN-QDs microrings, mode visualization theory in multilayer neural network, architecture of the spectral prediction network (SN), generation of the illumination pattern, data preprocessing, training of the spectral prediction network (SN), training of the tandem neural network (PDF)

## Notes

Ng, W. K.; Raziman, T. V.; Saxena, D.; Molkens, K.; Tanghe, I.; Xuan, Z.; Geiregat, P.; Van Thourhout, D; Barahona, M.; Sapienza, R. *Mode visualization and control of complex lasers using neural networks*, 2024, arXiv:2407.03815, arXiv preprint 10.48550/arXiv.2407.03815 (accessed September 1, 2025).
