# Supplementary material for: Mode Visualization and Control of Complex Lasers Using Neural Networks
Source: ACS Photonics. 2025 Sep 9;12(10):5774–81. doi: 10.1021/acsphotonics.5c01710 (PMC12532367; doi:10.1021/acsphotonics.5c01710)
Supplement: Supplementary file 1 [file ph5c01710_si_001.pdf]

# Supplementary information: Mode visualisation and control of complex lasers using neural networks

Wai Kit Ng,[1] T. V. Raziman,[1,2] Dhruv Saxena,[1] Korneel Molkens,[3,4,5] Ivo Tanghe,[3,4,5] Zhenghe Xuan,[1] Pieter Geiregat,[3,4] Dries Van Thourhout,[4,5] Mauricio Barahona,[2] and Riccardo Sapienza[1]

[1]*Blackett Laboratory, Department of Physics, Imperial College London, London, SW7 2BW, UK*

[2]*Department of Mathematics, Imperial College London, London, SW7 2AZ, UK*

[3]*Physics and Chemistry of Nanostructures (PCN), Ghent University, Krijgslaan 281-S3, B9000 Gent, Belgium*

[4]*Center for Nano- and Biophotonics, Ghent University, 9052 Ghent, Belgium*

[5]*Photonics Research Group, Ghent University - imec, Technologiepark-Zwijnaarde 126, 9052 Ghent, Belgium*

## CONTENTS

Note S1. Characteristic of SiN-QDs microrings S1

Note S2. Mode visualisation theory in multi-layer neural network S4

Note S3. Architecture of the spectral prediction network (SN) S6

Note S4. Generation of the illumination pattern S8

Note S5. Data preprocessing S10

Note S6. Training of the spectral prediction network (SN) S11

Note S7. Training of the tandem neural network S13

Supplementary References S19

**Note S1: Characteristic of SiN-QDs microrings**

The CdSe/CdS quantum dots (QDs) in a vertical SiN/QD/SiN stack structure supply gain to the microring lasers. The gain spectra of the CdSe/CdS QDs through transient absorption measurements are shown in Figure S1(a). By structurally confining the emission, a standalone 10 $\mu$m microring lases efficiently under uniform illumination, with a few different lasing peaks corresponding to different transverse and longitudinal whispering gallery modes (WGMs) (Figure S1(b)).

The 10×10 microring array presented in this work was designed and fabricated with slight variations in ring diameters and ring-to-ring gaps (See Methods for the selection of lasing structure). The rings were designed to be $\sim$ 10 $\mu$m in diameter with ring-to-ring gaps ranging from 0 – 240 nm and a fixed 2 $\mu$m width. The weak coupling from the disordered coupling strengths and detuned resonance frequencies across the array results in a complex lasing response (see Figure 1(b)). The statistical distributions of the ring diameters and gaps are shown in Figure S1(c), (d).

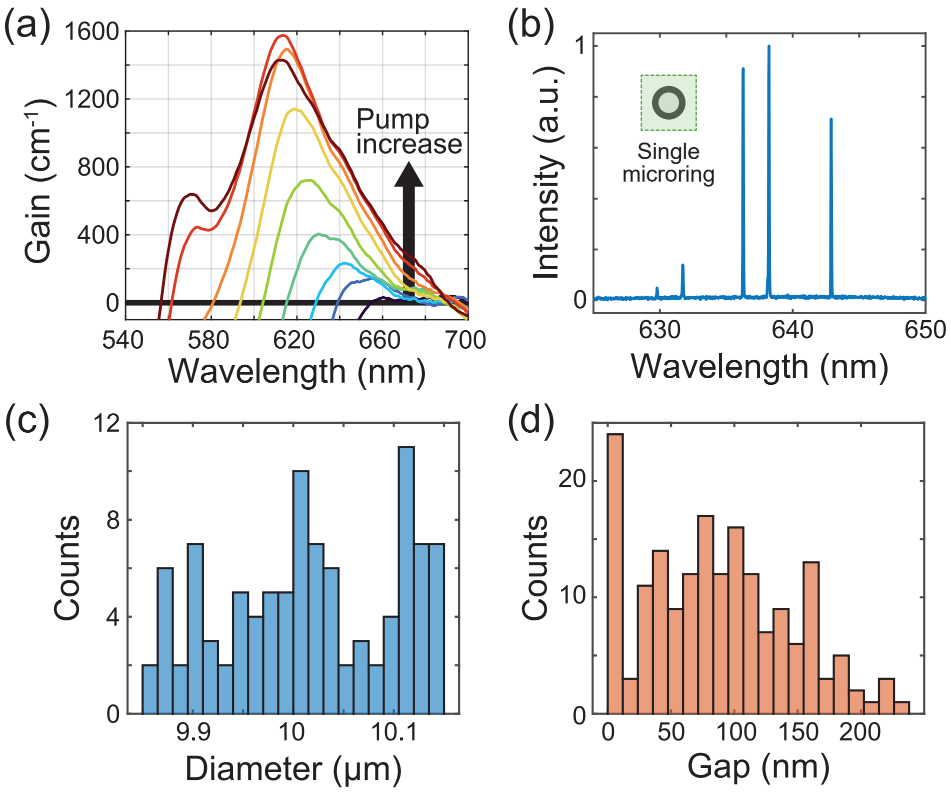


Figure S1. **Characterisations of the hybrid QD-SiN microring array.** (a) The gain spectrum of the CdSe/CdS quantum dots measured via transient absorption at 3 ps delay under different pump fluences [44]. (b) The emission spectrum of a standalone single microring laser with 10 $\mu$m diameter and 2 $\mu$m width. Multiple lasing modes are supported in the ring. (c, d) The statistical distributions of the (c) ring diameters and (d) ring-to-ring gaps in the 10×10 disorderly coupled microring array.

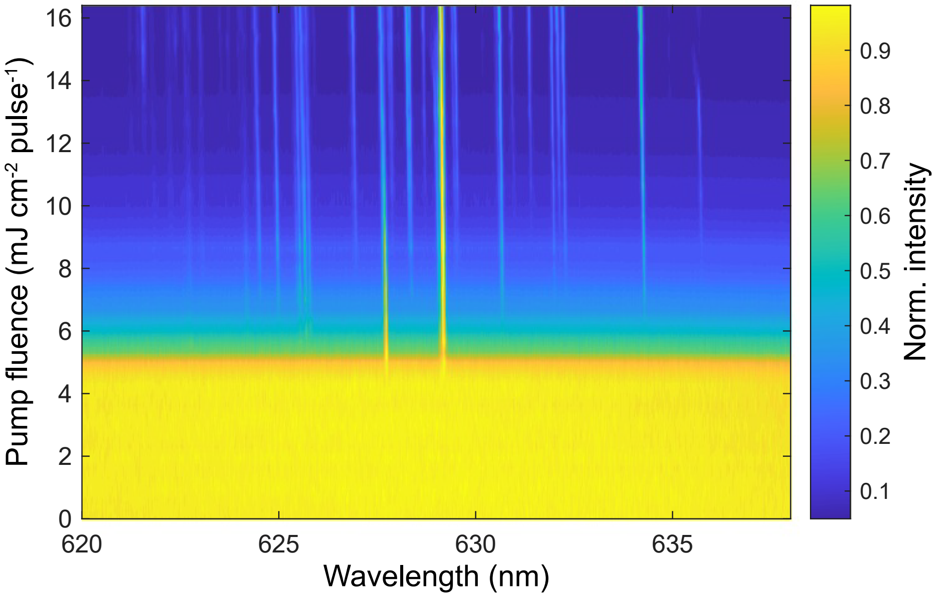


Figure S2. **Power-dependent lasing spectra of the hybrid QD-SiN microring array.** The normalised intensity spectra of the 10×10 disorderly coupled hybrid QD-SiN microring array under uniform excitation, showing mode enhancement and suppression as the pump increases.

**Note S2: Mode visualisation theory in multi-layer neural network**

In a single-layer neural network, the neural network architecture is mathematically written as

$$\mathbf{M} = \sigma(\mathbf{W} \cdot \mathbf{P} + \mathbf{B}), \tag{1}$$

where $\mathbf{P} = [p_1, ..., p_I]$ is the input layer and $\mathbf{M} = [m_1, ..., m_M]$ is the output layer of the neural network. The matrix $\mathbf{W}$ is the weight matrix describing the connection between $\mathbf{P}$ and $\mathbf{M}$, with the extra biases $\mathbf{B} = [b_1, ..., b_M]$. The mode visualisation theory for a single-layer neural network is described in the main text. In a 2-layer neural network, the complete architecture (without regularisations) can be written in the form of simultaneous equations,

$$\begin{cases} \mathbf{M} = \sigma_2(\mathbf{W}'' \cdot \mathbf{H} + \mathbf{B}'') \\ \mathbf{H} = \sigma_1(\mathbf{W}' \cdot \mathbf{P} + \mathbf{B}') \end{cases}, \tag{2}$$

where $\mathbf{W}'$ and $\mathbf{W}''$ are the weight matrices for the input-hidden and hidden-output layer connections. The activation functions $\sigma_1$ and $\sigma_2$ are the nonlinear functions applied to their corresponding layer. These simultaneous equations are equivalent to adding a layer $\mathbf{M}$ after the single-layer network with input $\mathbf{P}$ and output $\mathbf{H}$. Thus, the following 2-layer derivation can also be extended to N-layer neural networks by adding a layer to a (N-1)-layer neural network.

In a 2-layer neural network, for a particular output node $m_i$, the sum of the connections from the input layer to $m_i$ is

$$m_i = \sigma_2(\sum_k \mathbf{W}''_{\mathbf{ik}} \sigma_1(\sum_j \mathbf{W}'_{\mathbf{kj}}\, p_j + b'_k) + b''_i). \tag{3}$$

When considering the change of mode $m_i$ with respect to a spatial profile pixel $p_j$, the partial derivative $\frac{\partial m_i}{\partial p_j}$ can be expressed as

$$\begin{aligned} \frac{\partial m_i}{\partial p_j} &= \frac{\partial[\sigma_2(\sum_k \mathbf{W}''_{\mathbf{ik}} \sigma_1(\sum_j \mathbf{W}'_{\mathbf{kj}}\, p_j + b'_k) + b''_i)]}{\partial p_j} \\ &= \frac{\partial \sigma_2(u_i)}{\partial u_i} \cdot \sum_k \mathbf{W}''_{\mathbf{ik}} [\frac{\partial \sigma_1(v_k)}{\partial v_k} \cdot \frac{\partial(\sum_j \mathbf{W}'_{\mathbf{kj}}\, p_j + b'_k)}{\partial p_j}] \\ &= \frac{\partial \sigma_2(u_i)}{\partial u_i} \cdot \sum_k \mathbf{W}''_{\mathbf{ik}} [\frac{\partial \sigma_1(v_k)}{\partial v_k} \cdot \mathbf{W}'_{\mathbf{kj}}], \end{aligned} \tag{4}$$

in which the variables $u$ and $v$ are the dummy variables where $u_i = \sum_k \mathbf{W}''_{\mathbf{ik}} \sigma_1(\sum_j \mathbf{W}'_{\mathbf{kj}}\, p_j + b'_k) + b''_i$ and $v_k = \sum_j \mathbf{W}'_{\mathbf{kj}}\, p_j + b'_k$.

As in a single-layer neural network with different sets of values of $p_i$ $(i \neq j)$, these partial derivative terms are different because the lasing behaviour of mode $m_i$ is also dependent on the gain/loss distribution in proximity due to mode competition. The partial derivative terms (with local activation functions), however, are still constant across each pixel $p_j$ for the same target mode $m_i$. To compare the contributions between pixels for the same mode, we assume the system is populated close to the threshold of mode $m_i$ such that the small increase in the pixel value $p_j$ can lead to the activation of $\sigma_1$ and $\sigma_2$ (in the hidden layer and output layer respectively) if the pixel $p_j$ is important to the mode. Both the partial derivative terms are then not critical factors as they will remain at a similar level for different pixels $p_j$. The contributions of the spatial profile pixel $p_j$ to mode $m_i$, therefore, can be estimated by

$$\frac{\partial m_i}{\partial p_j} \sim \sum_k \mathbf{W''_{ik}} \mathbf{W'_{kj}} = \mathbf{W_{ij}}, \tag{5}$$

where $\mathbf{W} = \mathbf{W''} \cdot \mathbf{W'}$ is the equivalent weight matrix connecting input and output layers. Hence, the spatial gain profile of mode $m_i$ can be estimated by the $i^{th}$ row of the equivalent weight matrix $\mathbf{W}$.

### Note S3: Architecture of the spectral prediction network (SN)

The spectral prediction network (SN) is a densely connected artificial neural network (or MLP) with 2 hidden layers, as shown in Figure S3. Each pixel in an excitation pattern corresponds to a node in the input layer, and the input layer is connected to 2 hidden layers with 1024 and 64 nodes respectively. The processed information is propagated to the output layer with each node corresponding to a mode in the lasing spectra (one-hot encoded). For the 10×10 microring array, the excitation beam is patterned in a 30×26 grid that covers the entire array, with a total of 780 input pixels that can be switched on and off individually. By examining multiple lasing spectra, there are a total of 28 possible lasing modes identified.

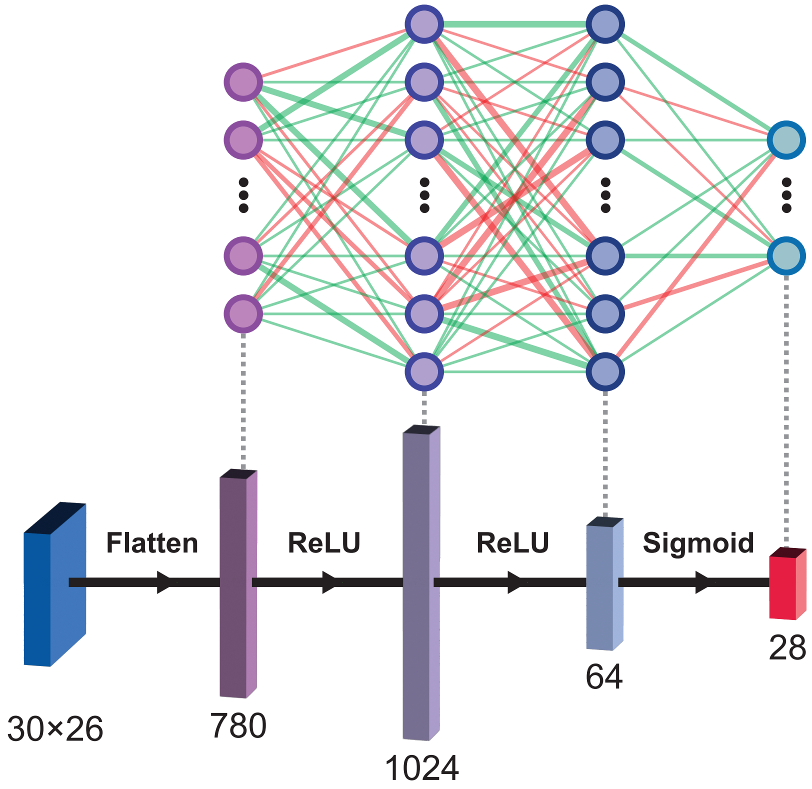


Figure S3. **Architecture of the spectral prediction network.** The 2-layer multi-label classification neural network model for mode visualisation. For the input and hidden layers (except the last hidden layer), a ReLU activation function is used. A sigmoid activation function is applied at the last hidden layer to produce a one-hot encoded mode spectral profile. The number of nodes in each layer is shown at the bottom.

## Note S4: Generation of the illumination pattern

The illumination patterns are Perlin noise patterns randomly generated using the fractal Brownian motion (fBm) technique. The pattern generation process is illustrated in Figure S4. A set of white noise patterns with various spatial frequencies (different octaves) are first generated. A single pattern is then created by summing the noises in different octaves, with higher spatial frequencies that are lower in amplitude. The resulting overall pattern, called "Perlin noise", shows some degree of clustering of pixels in random shapes to have a closer match with the gain distribution of photonic modes in general. The created Perlin noise is then binarised into black-and-white patterns for the patterned illumination using DMD. Since the spatial gain profiles of the modes are more likely to have low spatial frequency features across the photonic structure, training the MLP with Perlin noise patterns can reduce the amount of sampling required for more efficient training.

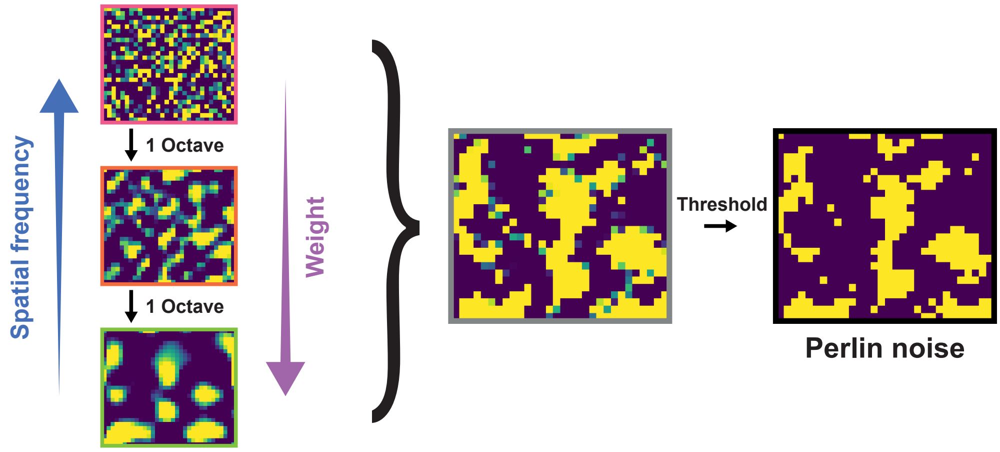


Figure S4. **The generation of Perlin noise patterns.** By combining multiple white noise patterns with different spatial frequencies and weights, the resulting binarised pattern resembles cluster features similar to those of the mode distributions in photonic cavities.

**Note S5: Data preprocessing**

Before feeding the data for the training of neural networks, the experimentally collected spectral data are pre-processed to simplify the classification task. Due to the photobleaching of the QDs gain under long illumination, the lasing spectra gradually offset across the data collection process. To correct for the shift of the emission spectrum, the spectrum of a reference excitation pattern (full illumination pattern) is collected for every 500 data points during the data collection. The data are then wavelength-corrected based on the shift between the reference spectra. After that, the background noise is subtracted from the wavelength-corrected spectra before we perform peak detection. The mode bins are extracted from the sum of all the collected spectra, with the tolerance range set to $\pm0.12$ nm (or the midpoint between two bins if the separation $< 0.12$ nm), to avoid mislabelling the modes across the datasets with offsets. For each spectrum, the detected peaks are then sorted into mode bins and converted into one-hot encoded mode data, which takes the form of a vector that only contains binary information on the existence of the modes (1 for lasing modes, 0 otherwise), to simplify the task in a multi-label classification problem.

**Note S6: Training of the spectral prediction network (SN)**

A set of 7000 randomly generated Perlin noise patterns is illuminated on the sample sequentially to obtain their spectra under a fixed excitation fluence. While the sample is partially illuminated, the lasing threshold of the sample varies, and the total energy delivered to the sample also depends on the area of the pattern. After testing with a small set of training patterns and different pump fluences, the pump with a fluence of 10.24 mJ $cm^{-2}$ $pulse^{-1}$ is chosen for both training and inference. This ensures that the system will lase under the majority of excitation patterns while keeping them not far from the lasing threshold. The lasing spectra collected from the pattern illuminations are first processed to extract the position and state (on or off) of the lasing peaks to simplify each lasing spectrum into a one-hot encoded $(28 \times 1)$ mode vector. The 7000 pairs of mode vectors and patterns are randomly split into sets of 5950 (85%) as a training dataset and 1050 (15%) as a test dataset. The test dataset is untouched during the training process, and it is only used to evaluate the performance of the model. During the backpropagation training process, the connection weights between the nodes (weight elements in $\mathbf{W_{HO}}$, $\mathbf{W_{HH}}$ and $\mathbf{W_{IH}}$) and the biases are adjusted based on the binary cross-entropy loss function calculated from the batches (batch size = 256) of the training dataset using the Adam optimisation algorithm (learning rate = 0.001). In each hidden layer, an L2 regulariser (with a decay rate of 0.001) is applied for ridge regression optimisation. This process is repeated until the network is well-trained and optimised, such that no further improvements can be made by updating the parameters when evaluating the test dataset. The evolution of the loss function and the corresponding prediction accuracies during the training process are monitored after each epoch, as shown in Figure S5. In total, 213 epochs have been trained. The prediction accuracy rapidly increased during the first 20 epochs and plateaued, with a 93.68% prediction accuracy for the test dataset.

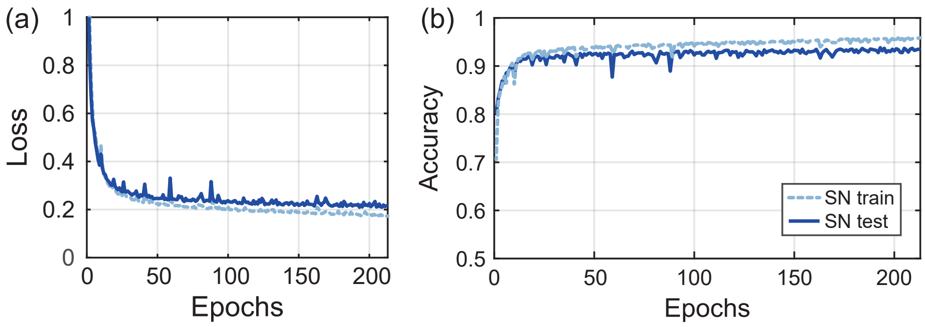


Figure S5. **The evolution of the spectral prediction network performance.** The evolution of the (a) loss and (b) prediction accuracy (binary accuracy) of the spectral prediction network during the backpropagation training.

**Note S7: Training of the tandem neural network**

The tandem neural network (TNN) model is initialised with an untrained control network (CN) and a well-trained spectral prediction network (SN) joined in series, as shown in Figure S6. The unique architecture of TNN with two feedforward MLPs offers the advantage of breaking down the complex inverse problem into relatively simple models and allowing the training to be done with conventional, basic optimisation methods. The SN is inherited from the MLP model used for mode visualisation. The CN is a different MLP with the lasing modes as input, mode profile pixels as output, and 3 hidden layers (64, 1024, and 1024 nodes respectively) in between to solve the more challenging inverse problem.

The SN is pre-trained with the 5950 sets of experimental data in the way described in the Note S6. During the TNN training, only the weights and biases in CN are updated, and the SN is treated as an untrainable network. The same training dataset used for the SN training is also used to train the TNN. When optimising the network to achieve good predictions for both the existence of spectral mode and the excitation pattern, the performance and the loss of the model are evaluated in the whole TNN. To achieve a realistic excitation pattern prediction from the target mode, the training takes into consideration both the spectral and spatial accuracies with an assigned complex loss function,

$$\begin{aligned} \text{loss} = &-\frac{1}{N}\sum_{n=1}^{N}\sum_{m=1}^{M}\left[S_{nm}\log S'_{nm} + (1-S_{nm})\log(1-S'_{nm})\right] \\ &+ \alpha \cdot \frac{1}{N}\sum_{n=1}^{N}\sum_{i=1}^{I}(P_{ni}-P'_{ni})^2, \end{aligned} \tag{6}$$

where $N = 5950$ is the size of the training dataset, $M = 28$ is the total number of modes, and $I = 780$ is the number of pixels in the patterns. $S_{nm}$ ($S'_{nm}$) and $P_{ni}$ ($P'_{ni}$) are the probabilities of the spectral mode and the spatial pattern pixel in the ground truth (prediction), respectively. The first term in this complex loss function is the binary cross-entropy function of the spectra, which is the same as the loss function in the SN training for mode visualisation. The second term is the mean-squared error function of the predicted excitation patterns. The constant $\alpha$ is a hyperparameter to optimise the model more towards either pattern similarities or mode accuracy. Here, the value of $\alpha = 0.25$ was chosen by brute-force search to obtain a good mode prediction accuracy while keeping the excitation pattern physical. The performance

evolution of the tandem neural network during backpropagation training is shown in Figure S7. Similar to the evolution in SN training (Figure S5), the accuracy of the model converges quickly, reaching an accuracy of around 72% after 200 epochs.

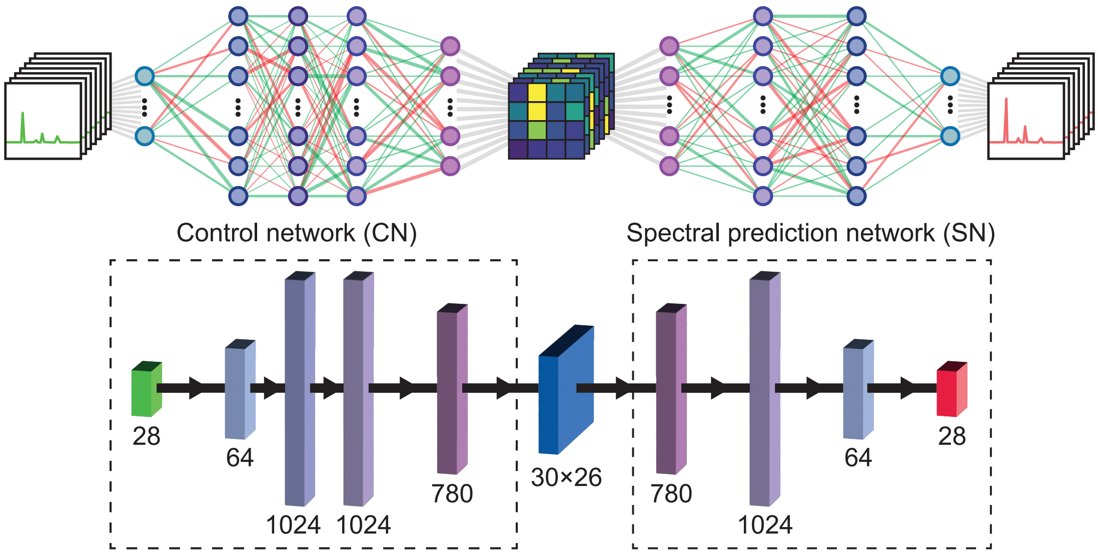


Figure S6. **Architecture of the tandem neural network.** The tandem neural network (TNN) consists of two feedforward MLPs (CN and SN). The number of nodes in each layer is shown at the bottom.

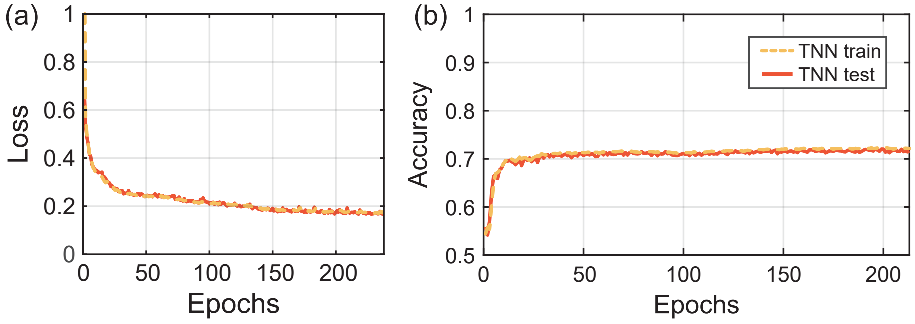


Figure S7. **The evolution of the tandem neural network performance.** The evolution of the (a) loss and (b) prediction accuracy (binary accuracy) of the tandem neural network during the backpropagation training, with the loss function considering both the spectral and spatial accuracies.

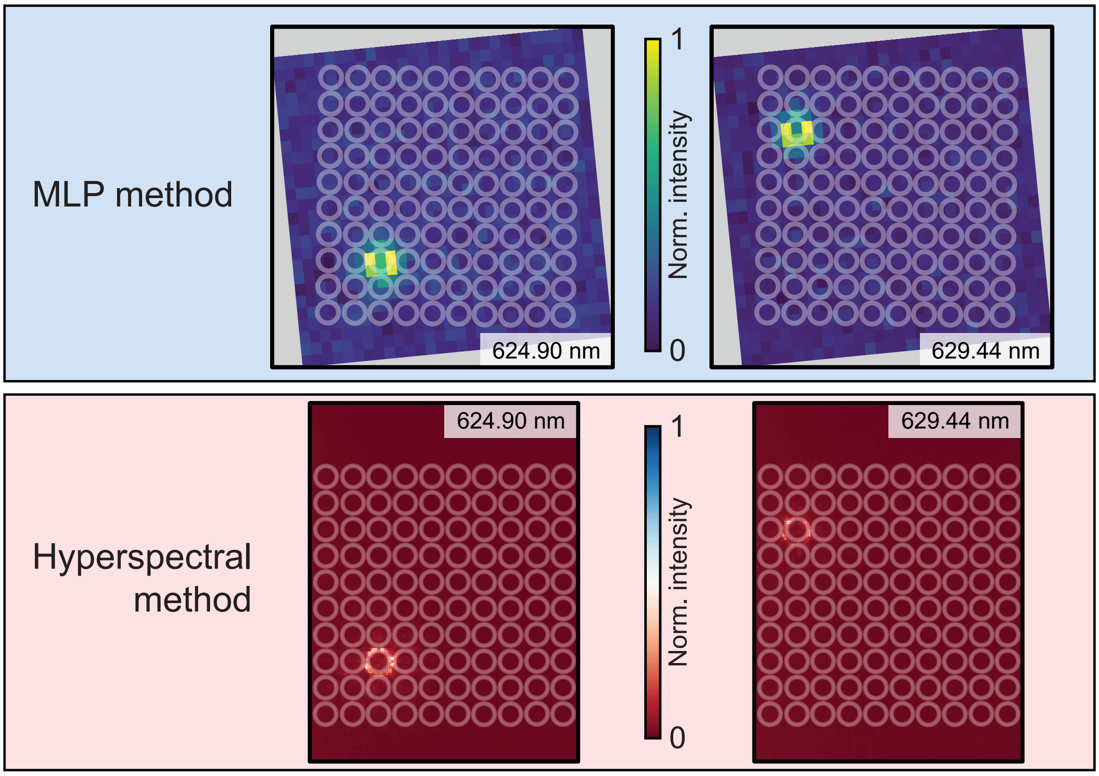


Figure S8. **The comparison between mode visualisation methods:** the machine learning MLP method in this study (blue) and the hyperspectral imaging method (red). The microring array structure is overlaid on the profiles to show the position of the mode relative to the array. For the coupled microring system with short waveguiding elements, similar gain profiles of different modes on the coupled microring system are revealed using two completely different methods.

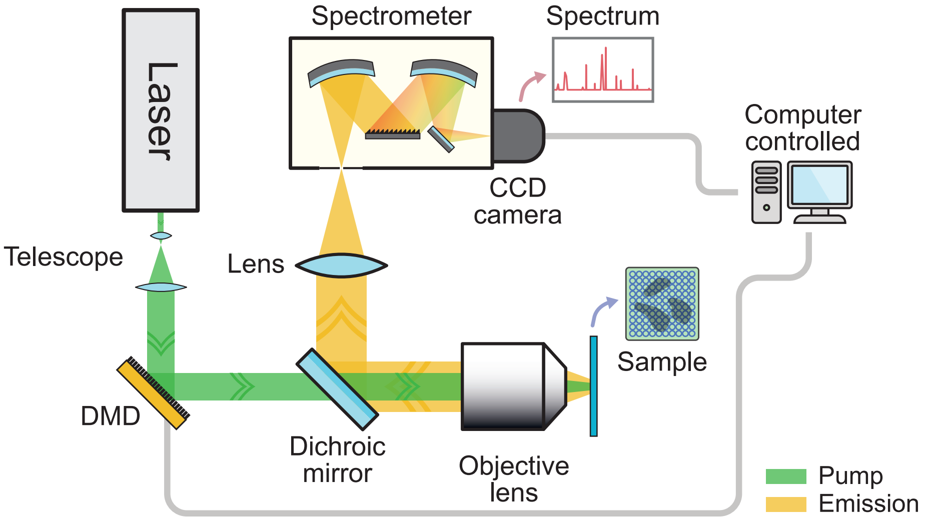


Figure S9. **Lasing spectroscopy with selective excitation.** The experimental setup for the collection of the lasing spectra from the coupled microring array. The excitation patterns are projected on the sample through a DMD. The lasing spectra are then collected by a spectrometer.

## SUPPLEMENTARY REFERENCES

[44] S. Bisschop, P. Geiregat, T. Aubert, and Z. Hens, The Impact of Core/Shell Sizes on the Optical Gain Characteristics of CdSe/CdS Quantum Dots, ACS Nano **12**, 9011 (2018).
